# Supplementary material for: Health Systems Determinants of Delivery and Uptake of Maternal Vaccines in Low- and Middle-Income Countries: A Qualitative Systematic Review
Source: Vaccines (Basel). 2023 Apr 19;11(4):869. doi: 10.3390/vaccines11040869 (PMC10144938; doi:10.3390/vaccines11040869)
Supplement: Supplementary file 1 [file vaccines-11-00869-s001.zip › Supplementary Material/S1 Table.docx]

**Table S1.** Literature search strategy for qualitative systematic review

| **Query** | **Key terms** |
| --- | --- |
| 1 | Maternal OR antenatal OR prenatal OR pregnancy OR trimester OR “maternal health services” OR “maternal health” |
| 2 | Vaccine OR immunization OR “immunization schedule” OR “immunization programs” OR “maternal vaccine” OR “vaccine during pregnancy” |
| 3 | #1 AND #2 |
| 4 | “Respiratory syncytial virus vaccine” OR “COVID 19 vaccine” OR “Cytomegalovirus vaccine” OR “Ebola virus vaccine” OR “Influenza vaccine” OR “Diphtheria tetanus acellular pertussis vaccine” OR “Tetanus vaccine” OR “pneumococcal vaccine” OR “oral Poliovirus vaccine” OR “Streptococcus Agalactiae” OR “Meningococcal vaccine” OR “Herpes simplex virus vaccine” OR “Group B Streptococcus” |
| 5 | #3 AND #4 |
| 6 | “Mass vaccination” OR “vaccine coverage” OR “vaccine introduction” OR “immunization communication” OR “immunization performance” OR “vaccine implementation” OR “vaccine introduction” OR “vaccine uptake” OR “immunization access” OR “vaccine supply” OR “vaccine distribution” OR acceptability OR “program legislation” OR “community and partnership” OR “program finance” OR “program management” OR “immunization program organization” OR “immunization program administration” |
| 7 | "Facilities and Services Utilization” OR "Delivery of Health Care" OR "Health system" OR "global health" OR "health information systems" OR "health financing" OR governance OR "policy" OR "health workforce" OR "healthcare personnel" OR "medical technologies" OR “medical products” OR "health systems strengthening" OR "health systems performance" OR "integrated health systems” OR "primary health care" OR "primary health service" OR “health system building blocks” OR “health system function” OR “health system capacity” |
| 8 | #6 OR #7 |
| 9 | #5 AND #8 |
| 10 | "Deprived countries" OR "developing countries" OR "less developed country" OR "low gross national" OR “low income” OR “middle income” OR LMIC OR LLMIC OR "low income countries" OR "middle income country" OR "middle income economies" OR "poor economy" OR "third world" OR "transitional country" OR "under developed economies" |
| 11 | Afghanistan OR Albania OR Algeria OR “American Samoa” OR Angola OR Armenia OR Azerbaijan OR Bangladesh OR Belarus OR Byelarus OR Belorussia OR Belize OR Benin OR Bhutan OR Bolivia OR Bosnia OR Botswana OR Brazil OR Bulgaria OR Burma OR “Burkina Faso” OR Burundi OR “Cabo Verde” OR “Cape verde” OR Cambodia OR Cameroon OR “Central African Republic” OR Chad OR China OR Colombia OR Comoros OR Comores OR Comoro OR Congo OR “Costa Rica” OR “Côte d'Ivoire” OR Cuba OR Djibouti OR Dominica OR “Dominican Republic” OR Ecuador OR Egypt OR “El Salvador” OR Eritrea OR Ethiopia OR Fiji OR Gabon OR Gambia OR Gaza OR “Georgia Republic” OR Georgian OR Ghana OR Grenada OR Grenadines OR Guatemala OR Guinea OR “Guinea Bisau” OR Guyana OR Haiti OR Herzegovina OR Hercegovina OR Honduras OR India OR Indonesia OR Iran OR Iraq OR Jamaica OR Jordan OR Kazakhstan OR Kenya OR Kiribati OR Korea OR Kosovo OR Kyrgyz OR Kirghizia OR Kirghiz OR Kirgizstan OR Kyrgyzstan OR “Lao PDR” OR Laos OR Lebanon OR Lesotho OR Liberia OR Libya OR Macedonia OR Madagascar OR Malawi OR Malay OR Malaya OR Malaysia OR Maldives OR Mali OR “Marshall Islands” OR Mauritania OR Mauritius OR Mexico OR Micronesia OR Moldova OR Mongolia OR Montenegro OR Morocco OR Mozambique OR Myanmar OR Namibia OR Nepal OR Nicaragua OR Niger OR Nigeria OR Pakistan OR Palau OR Panama OR “Papua New Guinea” OR Paraguay OR Peru OR Philippines OR Phillippines OR Philipines OR Phillipines OR Principe OR Romania OR Rwanda OR Ruanda OR Samoa OR “Sao Tome” OR Senegal OR Serbia OR “Sierra Leone” OR “Solomon Islands” OR Somalia OR “South Africa” OR “South Sudan” OR “Sri Lanka” OR “St Lucia” OR “St Vincent” OR Sudan OR Suriham OR Suriname OR Swaziland OR Syria OR “Syrian Arab Republic” OR Tajikistan OR Tadzhikistan OR Tadjikistan OR Tadzhik OR Tanzania OR Thailand OR Timor OR Togo OR Tonga OR Tunisia OR Turkey OR Turkmen OR Turkmenistan OR Tuvalu OR Uganda OR Ukraine OR Uzbek OR Uzbekistan OR Vanuatu OR Vietnam OR “West Bank” OR Yemen OR Zambia OR Zimbabwe |
| 12 | #10 OR #11 |
| 13 | #9 AND #12 |
| FILTERS | ENGLSH + FULL TEXT + HUMAN |
