# Supplementary material for: Health Systems Determinants of Delivery and Uptake of Maternal Vaccines in Low- and Middle-Income Countries: A Qualitative Systematic Review
Source: Vaccines (Basel). 2023 Apr 19;11(4):869. doi: 10.3390/vaccines11040869 (PMC10144938; doi:10.3390/vaccines11040869)
Supplement: Supplementary file 1 [file vaccines-11-00869-s001.zip › Supplementary Material/S2 Table.docx]

**Table S2.**  Summary of data extracted from studies included in this review.

| **No.** | **Author** | **Year published** | **Title** | **Country/ies** | **Vaccine** | **Participants** | **Health systems determinants of delivery** | **Health systems determinants of uptake** |
| --- | --- | --- | --- | --- | --- | --- | --- | --- |
| 1 | Otieno *et al.* | 2020 | Drivers and barriers of vaccine acceptance amongst pregnant women in Kenya | Kenya | Tetanus & Influenza | Pregnant women | Tetanus and influenza vaccines available and recommended | Healthcare workers main source of recommendation for vaccine |
| 2 | Otieno *et al.* | 2020 | Knowledge and attitudes towards influenza and influenza vaccination among pregnant women in Kenya | Kenya | Influenza | Pregnant women |  | Willingness to accept vaccine associated with belief that any maternal vaccines offered by public healthcare (‘government’) are trustworthy and beneficial |
| 3 | Nyiro *et al.* 2020 | 2020 | Implications of gestational age at antenatal care attendance on the successful implementation of a maternal respiratory syncytial virus (RSV) vaccine program in coastal Kenya | Kenya | Tetanus, to inform RSV | Women | Median initiation of ANC at 26 weeks  Only 10% of women attended >5 ANC  Issues with physical ANC booklets to track vaccine coverage | Education level significantly associated with pregnant women’s access of ANC  96% of women attended ANC between 24 and 36 weeks |
| 4 | Nganga  *et al*. | 2019 | Patient and provider perspectives on how trust influences maternal vaccine acceptance among pregnant women in Kenya | Kenya | Tetanus, general | Pregnant women; healthcare providers | Lack of vaccine education and communication by healthcare providers: workloads, time constraints | Trust in healthcare provider’s authority  Religious and cultural barriers to uptake  Cost barrier in rural context  Cost and provider attitudes affect uptake |
| 5 | Otieno  *et al*. | 2021 | Decision-making process for introduction of maternal vaccines in Kenya, 2017–2018 | Kenya | General | Policy makers | WHO recommendations and funding availability drive decision-making for vaccines  Maternal immunisation policy borrowed from existing policies for other vaccines  Gap in implementation; top down, lack of communication to facility-level | Factors outside of policy landscape that affect uptake: religious beliefs, anti-vaccine campaigns |
| 6 | Chander  *et al*. | 2020 | Antenatal care providers’ attitudes and beliefs towards maternal vaccination in Kenya | Kenya | Tetanus, influenza | Healthcare providers | Restricted resources: logistical and human  Good availability of vaccines  Public facilities had good education material and vaccine supply  Healthcare providers not regularly updated on vaccination guideline changes | High levels of trust between pregnant women and providers |
| 7 | Bergenfield  *et al*. | 2018 | Provider perspectives on demand creation for maternal vaccines in Kenya | Kenya | Tetanus | Healthcare providers | Devolution: county-based issues for supply, cold-chain and financing  Limited acceptance of influenza vaccine among healthcare providers | Barrier to uptake: cost of ANC, distance to facility in rural areas  Decision-making: women have limited agency, reliance on relatives |
| 8 | Giduthuri  *et al*. | 2019 | Influenza vaccination of pregnant women: Engaging clinicians to reduce missed opportunities for vaccination | India | Influenza. Intervention: 2 strategic interactions with HCPs | Women; community members Healthcare providers | Vaccination records (logs) difficult to maintain by healthcare workers  Vaccine availability differs by economic class: “slum” compared to “middle class” |  |
| 9 | Giduthuri  *et al*. | 2021 | Antenatal influenza vaccination in urban Pune, India: clinician and community stakeholders’ awareness, priorities, and practices | India | Influenza | Women; community members; Healthcare providers | Lack of healthcare provider awareness for influenza vaccine recommendation  Lack of clear policy in facilities | Perception that payment for services equates to vaccine information sharing and informed consent to vaccines  Barrier to uptake: cost (in slum areas), knowledge of vaccine  Decision-making: reliance on relatives |
| 10 | Malik  *et al*. | 2020 | Health care providers perspectives about maternal immunization in Latin America | Argentina, Brazil, Honduras, Mexico, Peru | Tetanus and influenza | Healthcare providers | Lack of co-ordinated approach between private and public sectors: recommendation discrepancies  Lack of funding for research  Stockouts jeopardise trust in providers  Lack of training for physicians | Accessing care is a challenge in rural/marginalised areas  Many women experience long waiting times at facilities |
| 11 | Malik  *et al*. | 2020 | Pregnant women’s perspectives about maternal immunization in Latin America | Argentina, Brazil, Honduras, Mexico, Peru | Influenza | Pregnant women | Long waiting times a facilities  Understaffed  Vaccine shortages | Healthcare providers are trusted for vaccine recommendations  Bad attitude of providers and treatment of pregnant women barrier to uptake  Reliance on social media for vaccine information |
| 12 | Alvarez  *et al* | 2020 | Enablers and barriers of maternal and neonatal immunization programs in Latin America | Latin America: Argentina, Brazil, Honduras, Mexico, Peru | General | Healthcare providers decision makers, community members, pregnant women | Considerations for vaccine policy: political will, feasibility, technicalities  Top-down policy communication  Domestic pooled funding  Limited service provider and platform coordination | Incentive: cash offers for vaccine |
| 13 | Simas  *et al* | 2021 | “Saint Google, now we have information!”: a qualitative study on narratives of trust and attitudes towards maternal vaccination in Mexico City and Toluca | Mexico | General | Women | Experienced shortages; reliance on private care if public is saturated.  Dual reliance on public and private providers | Dimensions of trust: safety, information; health system  Relatives influence decision-making |
| 14 | Gonzalez-Block  *et al*. | 2020 | Influenza vaccination hesitancy in five countries of South America. Confidence, complacency and convenience as determinants of immunization rates | South America (Brazil, Paraguay, Chile, Peru, Uruguay) | Influenza | Pregnant women |  | Less than half of the pregnant women had been vaccinated in the last year  Confidence is the key factor among pregnant women for vaccine hesitancy across countries. |
| 15 | Praphasiri  *et al*. | 2017 | Do Thai Physicians Recommend Seasonal Influenza Vaccines to Pregnant Women? A Cross-Sectional Survey of Physicians' Perspectives and Practices in Thailand | Thailand | Influenza | Healthcare providers | Ministry of Health Guidelines unclear  Healthcare provider knowledge of guidelines, vaccine safety facilitates high vaccine coverage  Storage issues reported  Influenza not part of routine ANC monitoring | Vaccines provided free of charge |
| 16 | Kaoiean  *et al*. | 2019 | Predictors for influenza vaccination among Thai pregnant woman: The role of physicians in increasing vaccine uptake | Thailand | Influenza | Pregnant women and Healthcare providers | All health care providers knew about national policy for influenza vaccine during pregnancy, but only 28% stated that they were frequent recommenders. | Awareness of National policy in vaccination seemed to be associated with likelihood of vaccinating  Social demographic factors like age, income, employment status/education did NOT associate with uptake.  Those who received recommendations from HCP were twice more likely to receive vaccine |
| 17 | Wang  *et al*. | 2019 | Low awareness of influenza vaccination among pregnant women and their obstetricians: a population-based survey in Beijing, China | China | Influenza | Pregnant women; Healthcare providers | Healthcare provider willingness to recommend vaccine was very low, associated with higher professional title but younger HCP age  Unwillingness was associated with safety concerns and AEFI | Positive association with vaccine uptake and history of prior vaccination; perceived benefits and knowledge of influenza; provider recommendation; media coverage |
| 18 | Wang  *et al*. | 2021 | Acceptance of seasonal influenza vaccination and associated factors among pregnant women in the context of COVID-19 pandemic in China: a multi-center cross-sectional study based on health belief model | China | Influenza | Pregnant women |  | Acceptance significantly associated with provider and family-member recommendations  Low acceptance levels attributed to lack of knowledge of vaccine purpose and safety |
| 19 | Li *et al.* | 2018 | Identifying ways to increase seasonal influenza vaccine uptake among pregnant women in China: A qualitative investigation of pregnant women and their obstetricians | China | Influenza | Pregnant women, Healthcare providers | Lack of National influenza guidelines  Lack of provider awareness of global CDC and WHO guidelines  Provider reliance on non-pharmaceutical interventions | Lack of awareness and effectiveness of vaccine barrier to uptake  Decision-making: influenced by health ministry, family members, providers |
| 20 | Kfouri & Richtmann | 2013 | Influenza vaccine in pregnant women: immunization coverage and associated factors | Brazil | Influenza | Women | 97% of the sample had been vaccinated during pregnancy  78% informed during antenatal period | Uptake associated with awareness of vaccine benefit to neonate |
| 21 | Mendonza-Sassi  *et al*. | 2019 | Vaccination against influenza among pregnant women in southern Brazil and associated factors | Brazil | Influenza | Women | Most women vaccinated in their third trimester; majority had received it in their lifetime. Prenatal care split between public and private evenly; with 80% accessing care in first trimester.  Delivery in private care decreased vaccination coverage compared to public | Private sector charges for vaccine  Education level does seem to be a significant factor for influenza uptake  Delayed prenatal care associated with lower vaccine uptake |
| 22 | Simas  *et al*. | 2021 | ‘‘From my phone, I could rule the world”: Critical engagement with maternal vaccine information, vaccine confidence builders and post-Zika outbreak rumours in Brazil | Brazil | General; Zika | Pregnant women |  | Prenatal experience was at private facilities, but vaccinating would happen at public (preference)  Lack of trust around Zika vaccines: government mistrust, safety concerns.  The vaccination card/booklet is used for info reference and vaccine record |
| 23 | Carcelen *et al.* | 2020 | Perceptions and attitudes towards vaccination during pregnancy in a peri urban area of Lima, Peru | Peru | General; Zika | Pregnant women | Two thirds of women had been vaccinated during pregnancy | Most told about vaccinations during ANC; low vaccine hesitancy  Lack of vaccine information is main barrier to uptake.  Provider recommendation facilitates uptake |
| 24 | Garcia  *et al.* | 2020 | ‘‘The flu... is a little more complicated than a cold”: Knowledge, beliefs, and practices related to influenza and influenza vaccination among at-risk populations and health professionals in Peru | Peru | Influenza | Pregnant women | Pregnant women not perceived to be 'high risk group to decision-makers  Vaccine shortage/supply issues; late arrival; long waiting times | Barrier to uptake: providers themselves, particularly doctors, are vaccine hesitant |
| 25 | Varan  *et al* . | 2014 | Intention to accept Bordetella pertussis booster vaccine during pregnancy in Mexico City | Mexico | Tetanus, influenza, pertussis | Women |  | Most respondents attended ANC during third trimester.  Majority received recommendation for tetanus, but was lower for influenza.  Health Ministry, providers and media are sources of information |
| 26 | Honarvar  *et al*. | 2012 | Acceptance and rejection of influenza vaccination by pregnant women in southern Iran | Iran | Influenza | Pregnant women | Only pregnant women with a history of chronic disease have been included  in National Immunisation Programme | Social determinants were not NB factors that affect likelihood of vaccination  Uptake VERY low of influenza (6%): lack of knowledge and information |
| 27 | Laizer  *et al*. | 2021 | Challenges Experienced by Healthcare Workers on Maternal Tetanus Toxoid Vaccination in Kilimanjaro Region Tanzania | Tanzania | Tetanus | Healthcare providers | Unreliable Tetanus vaccine storage (interrupted power supply) leads to reliance on other sources that take long to procure (like gas)  Movement of vaccines from district to facility is a barrier; transportation issues.  Division of labour between nurses and doctors: lack of coordinated team-work | Follow-up doses jeopardised because of local cultural birth norms  Delayed ANC  Decision-making: spousal influence |
| 28 | Johm  *et al*. | 2021 | Factors influencing acceptance of vaccination during pregnancy in The Gambia and Senegal | Senegal, Gambia | Tetanus | Women | Vaccines not integrated in ANC (Senegal) | Some communities have sense of honour in being able to pay for services; reluctant to receive free vaccines at no cost  Need for sensitisation of community for new vaccines  High trust in providers |
| 29 | Giles  *et al*. | 2020 | Vaccine implementation factors affecting maternal tetanus immunization in low- and middle-income countries: Results of the Maternal Immunization and Antenatal Care Situational Analysis (MIACSA) project | Range of LMICs | Tetanus | Policy-makers; Healthcare providers; pregnant women | Policy and targets set at national level not necessarily known at facility level  EPI is the procurement platform for most countries  Nurses trained frequently |  |
| 30 | Anatea  *et al*. | 2018 | Determinants and perceptions of the utilization of tetanus toxoid immunization among reproductive-age women in Dukem Town, Eastern Ethiopia: a community-based cross-sectional study | Ethiopia | Tetanus | Women | Facility waiting times and staff treatment of patients affect delivery  60% had less than 4 ANC visits | Variables that significantly affected utilisation: education level, TV in the house, occupation status, place of birth, knowledge of Tetanus vaccine |
| 31 | Gebremedhin  *et al*. | 2020 | Tetanus Toxoid Vaccination Uptake and Associated Factors among Mothers Who Gave Birth in the Last 12 Months in Errer District, Somali Regional State, Eastern Ethiopia | Ethiopia | Tetanus | Women |  | Barriers to uptake include very long travel times to facilities and Low satisfactory knowledge of vaccine  Increased uptake with urban residency, short travel distance, maternal education |
| 32 | Arifin  *et al*. | 2021 | Regional disparities and their contribution to the coverage of the tetanus toxoid vaccine among women aged 15–49 years in Indonesia | Indonesia | Tetanus | Pregnant women | Coverage was associated with residence (rural/urban), wealth quintiles, health insurance, education level, age, employment status. | More likely to get vaccinated in rural areas  Compliance and trust in healthcare providers |
| 33 | Bishop  *et al*. | 2021 | An evaluation of an influenza vaccination campaign targeting pregnant women in 27 clinics in two provinces of South Africa, 2015 – 2018 | South Africa | Influenza | Pregnant women | Barriers to delivery: stock outs, cold chain management and waste  Did not affect staff functions for ANC  High coverage associated with provider training | High coverage associated with maternal education |
| 34 | Erazo  *et al* | 2020 | Knowledge, attitudes and practices on influenza vaccination during pregnancy in Quito, Ecuador | Ecuador | Influenza | Women | High ANC attendance: 80% attended over 4 visits  Increased ANC visits associated with vaccination during pregnancy  Reliance on self-reporting because women lack documentation | Knowledge of vaccine and perception of safety and effectiveness significantly higher in vaccinated women  Lack of healthcare provider recommendation is main barrier to uptake |
| 35 | Nguyen  *et al*. | 2021 | Acceptance and willingness to pay for COVID-19 vaccines among pregnant women in Vietnam | Vietnam | COVID-19 | Women |  | Education associated with vaccine acceptance: higher education more likely to vaccinate |
| 36 | Fleming  *et al*. | 2018 | Implementation of maternal influenza immunization in El Salvador: Experiences and lessons learned from a mixed-methods study | El Salvador | Influenza | Community members; Healthcare providers | Primary delivery through vaccination weeks  Notable increased acceptance of vaccine by HCPs over time, which improved acceptance by community | Public facilities offer vaccine free of charge; private providers often refer to them  Providers trusted  Decision-making: reliance on family and community  Crime major barrier to access |
| 37 | Fleming  *et al*. | 2019 | Maternal immunization in Malawi: A mixed methods study of community perceptions, programmatic considerations, and recommendations for future planning | Malawi | Influenza and tetanus | Healthcare providers; policy makers, community members; pregnant women. | Local laws aimed to increase health coverage, like laws requiring tetanus vaccination, incentivised  Lack of resources to cope with waste  Information documented at facility & community level, but not shared between sites: difficult to track vaccination and doses | Community health workers main source of info, followed by HCPs  Concern that injectable contraceptive concerns overlap/confuse vaccine perceptions  Purchasing of vaccines by community members deems the vaccines safe  Religious affiliation and family opinions barrier to uptake |
| 38 | Kajungu  *et al*. | 2020 | Vaccines safety and maternal knowledge for enhanced maternal immunization acceptability in rural Uganda: A qualitative study approach | Uganda | Tetanus | Women and Healthcare providers | Vaccine only offered 2 days of the week, weekdays  Long waiting times  Lack of sufficient healthcare providers | women who use traditional healers and do not access facilities  Fear of mandatory HIV testing for ANC also wards women off.  High willingness to receive vaccines if sensitised to them |
| 39 | Lohiniva  *et al* | 2014 | A Qualitative Study of Vaccine Acceptability and Decision Making among Pregnant Women in Morocco during the A (H1N1) pdm09 Pandemic | Morocco | Influenza | Women |  | Distrust of vaccination intent: for-profit by manufacturers and fear of foreign interference  Barrier to uptake: lack of sufficient information from provider or no recommendation at all |
| 40 | Yaya  *et al.* | 2019 | Antenatal visits are positively associated with uptake of tetanus toxoid and intermittent preventive treatment in pregnancy in Ivory Coast | Ivory Coast | Tetanus | Pregnant women | 79% had adequate tetanus coverage (>2 doses) | Higher uptake was seen in group with higher education, younger in age, rural residency, at least 3 ANC visits. |
| 41 | Yaya  *et al*. | 2020 | Prevalence and predictors of taking tetanus toxoid vaccine in pregnancy: a cross-sectional study of 8,722 women in Sierra Leone | Sierra Leone | Tetanus | Women |  | Higher tetanus coverage associated with increased ANC, Higher wealth quintile, rural residency. |
| 42 | Larson Williams *et al.* | 2018 | ‘‘When you are injected, the baby is protected:” Assessing the acceptability of a maternal Tdap vaccine based on mothers’ knowledge, attitudes, and beliefs of pertussis and vaccinations in Lusaka, Zambia | Zambia | Tdap | Women |  | Limited knowledge and misconceptions around vaccines and associated diseases  community rumours fuel hesitancy  Barrier to uptake: partner/spousal hesitancy |
| 43 | Giles  *et al* | 2020 | Antenatal care service delivery and factors affecting effective tetanus vaccine coverage in low- and middle-income countries: Results of the Maternal Immunisation and Antenatal Care Situational analysis (MIACSA) project | Range of LMICs | Tetanus | Pregnant women; Healthcare providers | 20% of countries indicated ANC being funded partial by out-of-pocket payments | Imposing user fees was significantly associated with lower tetanus vaccine coverage |
| 44 | Arriola  *et al* | 2018 | Knowledge, attitudes, and practices about influenza vaccination among pregnant women and healthcare providers serving pregnant women in Managua, Nicaragua | Nicaragua | Influenza | Pregnant women; Healthcare providers | Most countries reported that ANC was dependent on national budget, and half of the countries benefit from external donor funding  Predominant reliance on passive reporting for diseases  Higher ANC visit recommendation was associated with higher vaccine coverage | Major barrier to uptake was unawareness of vaccine  Social demographic factors had no association with uptake  Less than half received recommendations from healthcare providers |
| 45 | Arriola  *et al* | 2016 | Factors associated with a successful expansion of influenza vaccination among pregnant women in Nicaragua | Nicaragua | Influenza | Pregnant women | Majority received influenza vaccine in the third trimester  Unavailability of vaccines at some facilities was a barrier to delivery  High healthcare provider belief that vaccines are effective, and most knew of the tetanus vaccine recommendation | No association of vaccine with social demographics |
| 46 | Li  *et al.* | 2020 | Factors affecting pregnant women's decisions regarding prenatal pertussis vaccination: A decision-making study in the nationwide Prenatal Pertussis Immunization Program in Taiwan | Taiwan | Tdap | Pregnant women | 71% of women had received vaccine  73% attended >4 ANC visits | Social demographics NOT associated with uptake  Uptake is associated with sufficient information provision around the vaccine  Provider recommendations influential to uptake |
| 47 | Wong  *et al*. | 2017 | Attitudes towards Zika screening and vaccination acceptability among pregnant women in Malaysia | Malaysia | Zika | Pregnant women |  | Willingness to take vaccine if recommended by healthcare provider  Decision-making Friends, family and spouses cited as major influences for vaccine recommendation |
| 48 | Toure *et al.* | 2022 | Facilitators and barriers to COVID-19 vaccination among healthcare workers and the general population in Guinea. | Guinea | Covid-19 | Pregnant women |  | Lack of trust/confidence in government limits vaccine acceptance.  Better vaccine uptake is associated with marital status (marriage), lower vaccine knowledge, higher income, being an older adult and being pregnant. |
| 49 | Amin *et al.* | 2022 | Trends and determinants of taking tetanus toxoid vaccine among women during last pregnancy in Bangladesh: Country representative survey from 2006 to 2019. | Bangladesh | Tetanus | Women of childbearing age | There is a decreasing trend in Tetanus coverage over the defined time period.  Coverage was better in those who utilised public services for maternal care in 2019. | Full coverage for Tetanus associated with higher education; urban residence; higher wealth quintile; use of immunization cards. |
| 50 | Asratie *et al.* | 2022 | Perception of risk regarding the use of COVID-19 vaccine among pregnant women in Motta town and Hulet Eji Enese district, northwest Ethiopia. | Ethiopia | Covid-19 | Pregnant women |  | Perceived risk of Covid-vaccine and lower acceptance associated with lower educational level; not utilising ANC services; long travel times to facilities; unplanned pregnancy. |
| 51 | Aynalem *et al.* | 2022 | Factors associated with willingness to take COVID-19 vaccine among pregnant women at Gondar town, Northwest Ethiopia: A multicenter institution-based cross-sectional study. | Ethiopia | Covid-19 | Pregnant women |  | Increased acceptance of vaccine associated with history of chronic illness; pregnancy; older age |
| 52 | Madubueze *et al.* | 2022 | Awareness, knowledge, risk perception and uptake of maternal vaccination  in rural communities of Ebonyi State, Nigeria | Nigeria | Tetanus | Women of childbearing age |  | Higher knowledge of Tetanus vaccine compared to other vaccines, although knowledge is still very low.  Information sources for vaccines are mainly healthcare providers.  Uptake significantly positively associated with marital status, but negatively associated with higher education level. |
| 53 | Chimukuche *et al.* | 2022 | Assessing Community Acceptance of Maternal  Immunisation in Rural KwaZulu-Natal, South Africa: A Qualitative Investigation | South Africa | General | Pregnant women  Healthcare providers  Community members | Vaccine administration viewed as the law, and delivery did not include vaccine information or engagement.  Vaccine stockouts mean Tetanus coverage is not comprehensive (often only receiving one dose and resulting missed opportunities when ANC is accessed) | Barriers to uptake include influence of women’s partners and community members through negative sentiments about ANC, and religious/cultural beliefs, Low knowledge of vaccine, low utilization of ANC, long waiting queues at facilities and long travelling distances |
| 54 | Belizán *et al.* | 2023 | Data collection systems for active safety surveillance of vaccines during pregnancy in low‑ and middle‑income countries: developing and piloting an assessment tool (VPASS) | Uganda, Rwanda, India, Zambia,  Burkina Faso, Mozambique | General | - | Data collection and capture barriers in Uganda: limited access to technology resources (Internet connectivity and data analysts)  Burkina Faso and Mozambique: decentralized data collection at health facilities limits effective use. Collection is paper-based, and limited resources for electronic data collection are barriers to implementation for vaccine safety surveillance. |  |
