# Supplementary material for: Health Systems Determinants of Delivery and Uptake of Maternal Vaccines in Low- and Middle-Income Countries: A Qualitative Systematic Review
Source: Vaccines (Basel). 2023 Apr 19;11(4):869. doi: 10.3390/vaccines11040869 (PMC10144938; doi:10.3390/vaccines11040869)
Supplement: Supplementary file 1 [file vaccines-11-00869-s001.zip › Supplementary Material/S3 Table.docx]

**Table S3.** Summary of quality appraisal of studies included in this review

| **Article details** | | | | **Appraisal Tool Questions [26,27]^a^** | | | | | | | | | | | | | **Overall quality appraisal** |
| --- | --- | --- | --- | --- | --- | --- | --- | --- | --- | --- | --- | --- | --- | --- | --- | --- | --- |
| **Article no.** | **Author** | **Year published** | **Study design** | **1** | **2** | **3** | **4** | **5** | **6** | **7** | **8** | **9** | **10** | **11** | **12** | **13** |  |
| 1 | Alvarez *et al.* | 2021 | Qualitative | Y | Y | Y | Y | Y | N | N | Y | Y | Y |  |  |  | Moderate |
| 2 | Alvarez *et al.* | 2020 | Mixed method | Y | Y | Y | Y | Y |  |  |  |  |  |  |  |  | High |
| 3 | Anatea *et al..* | 2018 | Cross-sectional | Y | Y | Y | Y | Y | Y | Y | Y |  |  |  |  |  | High |
| 4 | Arifin *et al.* | 2021 | Cross-sectional | Y | Y | Y | Y | Y | Y | Y | Y |  |  |  |  |  | High |
| 5 | Arriola *et al.* | 2018 | Cross-sectional | Y | Y | Y | Y | Y | Y | Y | Y | Y | Y |  |  |  | High |
| 6 | Arriola *et al.* | 2016 | Cross-sectional | Y | Y | Y | Y | Y | Y | Y | Y | Y | Y |  |  |  | High |
| 7 | Bergenfield *et al.* | 2018 | Qualitative | U | Y | Y | Y | Y | Y | Y | Y |  |  |  |  |  | Moderate |
| 8 | Bishop *et al.* | 2021 | Cross-sectional | Y | Y | Y | Y | Y | Y | Y | Y |  |  |  |  |  | High |
| 9 | Carceren *et al.* | 2020 | Qualitative | U | Y | Y | Y | Y | U | N | Y | Y | Y |  |  |  | Low |
| 10 | Chander *et al.* | 2020 | Quantitative | N | Y | Y | Y | Y | Y | Y | Y |  |  |  |  |  | Moderate |
| 11 | Erazo *et al.* | 2020 | Cross-sectional | Y | Y | Y | Y | Y | Y | Y | Y |  |  |  |  |  | High |
| 12 | Fleming *et al*. | 2018 | Mixed Method | Y | Y | Y | Y | Y |  |  |  |  |  |  |  |  | High |
| 13 | Fleming *et al*. | 2019 | Mixed Method | Y | Y | Y | Y | Y |  |  |  |  |  |  |  |  | High |
| 14 | Garcia *et al.* | 2020 | Qualitative | U | Y | Y | Y | Y | N | N | Y | Y | Y |  |  |  | Moderate |
| 15 | Gebremedhin *et al*. | 2020 | Cross-sectional | Y | Y | Y | Y | U | Y | Y | Y |  |  |  |  |  | High |
| 16 | Giduthuri *et al.* | 2019 | Randomised control trial | Y | Y | Y | N | N | Y | Y | Y | N | Y | Y | Y | Y | Moderate |
| 17 | Giduthuri *et al.* | 2021 | Qualitative | U | Y | Y | Y | Y | N | N | Y | Y | Y |  |  |  | Low |
| 18 | Giles *et al.* | 2020 | Cross-sectional | U | Y | Y | Y | Y | U | Y | Y |  |  |  |  |  | Moderate |
| 19 | Giles *et al.* | 2020 | Mixed-method | Y | Y | Y | Y | Y |  |  |  |  |  |  |  |  | High |
| 20 | Gonzalez-Block *et al.* | 2020 | Quantitative | Y | Y | Y | Y | Y | Y | Y | Y |  |  |  |  |  | High |
| 21 | Honarvar *et al.* | 2012 | Cross-sectional | Y | Y | Y | Y | Y | Y | U | Y |  |  |  |  |  | High |
| 22 | Johm *et al.* | 2021 | Qualitative | Y | Y | Y | Y | Y | Y | U | Y | Y | Y |  |  |  | Moderate |
| 23 | Kajungu *et al.* | 2020 | Qualitative | Y | Y | Y | Y | Y | N | Y | Y | Y | Y |  |  |  | Moderate |
| 24 | Kaoiean *et al*. | 2019 | Cohort | Y | Y | Y | Y | Y | Y | Y | Y | Y | Y | Y |  |  | High |
| 25 | Kfouri *et al.* | 2013 | Quantitative descriptive | Y | Y | Y | Y | U | N | Y | Y |  |  |  |  |  | Low |
| 26 | Laizer *et al.* | 2021 | Qualitative | N | Y | Y | Y | Y | N | N | Y | Y | Y |  |  |  | Low |
| 27 | Larson Williams *et al.* | 2019 | Qualitative | Y | Y | Y | Y | Y | U | N | Y | Y | Y |  |  |  | Moderate |
| 28 | Li *et al.* | 2020 | Cross-sectional | Y | Y | Y | Y | Y | Y | U | Y | Y | Y |  |  |  | Moderate |
| 29 | Lohiniva *et al.* | 2014 | Qualitative | Y | Y | Y | Y | Y | U | Y | Y | Y | Y |  |  |  | Moderate |
| 30 | Malik *et al.* | 2020 | Qualitative | Y | Y | Y | Y | Y | N | N | Y | Y | Y |  |  |  | Moderate |
| 31 | Medonza-Sassi *et al.* | 2019 | Cross-sectional quantitative | Y | Y | Y | Y | Y | U | Y | Y |  |  |  |  |  | Moderate |
| 32 | Nganga *et al.* | 2019 | Qualitative | Y | Y | Y | Y | Y | Y | Y | Y | Y | Y |  |  |  | High |
| 33 | Nguyen *et al*. | 2021 | Cross-sectional | Y | U | Y | Y | Y | Y | Y | Y |  |  |  |  |  | Moderate |
| 34 | Nyiro *et al.* | 2020 | Quantitative | Y | Y | Y | Y | Y | Y | Y | Y |  |  |  |  |  | High |
| 35 | Otieno *et al*. | 2020 | Quantitative | Y | Y | Y | Y | Y | N | Y | Y |  |  |  |  |  | Moderate |
| 36 | Otieno *et al*. | 2020 | Quantitative | Y | Y | Y | Y | Y | Y | Y | Y |  |  |  |  |  | High |
| 37 | Otieno *et al*. | 2020 | Qualitative | Y | Y | Y | Y | Y | N | N | Y | Y | Y |  |  |  | Moderate |
| 38 | Praphasiri *et al*. | 2017 | Quantitative | Y | Y | Y | Y | N | N | Y | Y |  |  |  |  |  | Moderate |
| 39 | Li *et al.* | 2018 | Qualitative | U | Y | Y | Y | Y | U | N | Y | Y | Y |  |  |  | Low |
| 40 | Simas *et al.* | 2021 | Qualitative | U | Y | Y | Y | Y | N | N | Y | Y | Y |  |  |  | Low |
| 41 | Simas *et al.* | 2021 | Qualitative | U | Y | Y | Y | Y | N | N | Y | Y | Y |  |  |  | Moderate |
| 42 | Varan *et al.* | 2014 | Descriptive cross-sectional | Y | Y | Y | Y | Y | U | Y | Y |  |  |  |  |  | Moderate |
| 43 | Wang *et al*. | 2019 | Cross-sectional | Y | Y | Y | Y | Y | Y | Y | Y |  |  |  |  |  | High |
| 44 | Wang *et al*. | 2021 | Cross-sectional | Y | Y | Y | Y | N | U | Y | Y |  |  |  |  |  | Moderate |
| 45 | Wong *et al.* | 2017 | Cross-sectional | Y | Y | Y | Y | U | Y | Y | Y |  |  |  |  |  | Moderate |
| 46 | Yaya *et al.* | 2019 | Cross-sectional | Y | Y | Y | Y | Y | Y | Y | Y |  |  |  |  |  | High |
| 47 | Yaya *et al.* | 2020 | Cross-sectional | Y | Y | Y | Y | Y | Y | Y | Y |  |  |  |  |  | High |
| 48 | Toure *et al.* | 2022 | Mixed Method | Y | Y | Y | Y | Y |  |  |  |  |  |  |  |  | High |
| 49 | Amin *et al.* | 2022 | Cross-sectional | Y | Y | Y | Y | N | N | Y | Y |  |  |  |  |  | Moderate |
| 50 | Asratie *et al.* | 2022 | Cross-sectional | Y | Y | Y | Y | N | Y | Y | Y |  |  |  |  |  | High |
| 51 | Aynalem *et al.* | 2022 | Cross-sectional | Y | Y | Y | Y | N | Y | Y | Y |  |  |  |  |  | Moderate |
| 52 | Chimukuche *et al.* | 2022 | Qualitative | Y | Y | Y | Y | Y | N | N | Y | Y | Y |  |  |  | Moderate |
| 53 | Madubueze *et al.* | 2022 | Cross-sectional | Y | Y | Y | Y | N | N | Y |  |  |  |  |  |  | Moderate |
| 54 | Belizan *et al.* | 2023 | Qualitative | Y | Y | Y | Y | Y | Y | Y | Y | Y | Y |  |  |  | High |

*U= unclear; Y=Yes; N=No*

*^a^Questions for appraisal depend on stated study design*
